# Quantitative evaluation of oxygen metabolism in the intratumoral hypoxia: ^18^F-fluoromisonidazole and ^15^O-labelled gases inhalation PET

**DOI:** 10.1186/s13550-017-0263-6

**Published:** 2017-02-16

**Authors:** Tadashi Watabe, Yasukazu Kanai, Hayato Ikeda, Genki Horitsugi, Keiko Matsunaga, Hiroki Kato, Kayako Isohashi, Kohji Abe, Eku Shimosegawa, Jun Hatazawa

**Affiliations:** 1 Department of Nuclear Medicine and Tracer Kinetics, Osaka University Graduate School of Medicine, Suita, Japan; 2 Medical Imaging Center for Translational Research, Osaka University Graduate School of Medicine, Suita, Japan; 3 Department of Molecular Imaging in Medicine, Osaka University Graduate School of Medicine, Suita, Japan; 4 Department of Drug Metabolism & Pharmacokinetics, Osaka University Graduate School of Medicine, Suita, Japan; 5 Research Laboratory for Development, Shionogi & Co., Ltd.; Immunology Frontier Research Center, Osaka University, Suita, Japan

**Keywords:** PET, Fluoromisonidazole, Hypoxia, Blood flow, Oxygen consumption

## Abstract

**Background:**

Intratumoral hypoxia is one of the resistant factors in radiotherapy and chemotherapy for cancer. Although it is detected by ^18^F-fluoromisonidazole (FMISO) PET, the relationship between intratumoral hypoxia and oxygen metabolism has not been studied. The purpose of this study was to evaluate the intratumoral perfusion and oxygen metabolism in hypoxic regions using the rat xenograft model. Ten male Fischer rats with C6 glioma (body weight = 220 ± 15 g) were investigated with ^18^F-FMISO PET and steady-state inhalation method of ^15^O-labelled gases PET. The tumoral blood flow (TBF), tumoral metabolic rate of oxygen (TMRO_2_), oxygen extraction fraction (OEF), and tumoral blood volume (TBV) were measured under artificial ventilation with ^15^O–CO_2_, ^15^O–O_2_, and ^15^O–CO gases. Multiple volumes of interest (1-mm diameter sphere) were placed on the co-registered ^18^F-FMISO (3 h post injection) and functional ^15^O-labelled gases PET images. The TBF, TMRO_2_, OEF, and TBV values were compared among the three groups classified by the ^18^F-FMISO uptake as follows: group Low (L), less than 1.0; group Medium (M), between 1.0 and 2.0; and group High (H), more than 2.0 in the ^18^F-FMISO standardized uptake value (SUV).

**Results:**

There were moderate negative correlations between ^18^F-FMISO SUV and TBF (*r* = −0.56 and *p* < 0.01), and weak negative correlations between ^18^F-FMISO SUV and TMRO_2_ (*r* = −0.38 and *p* < 0.01) and ^18^F-FMISO SUV and TBV (*r* = −0.38 and *p* < 0.01). Quantitative values were as follows: TBF, (L) 55 ± 30, (M) 32 ± 17, and (H) 30 ± 15 mL/100 mL/min; OEF, (L) 33 ± 14, (M) 36 ± 17, and (H) 41 ± 16%; TMRO_2_, (L) 2.8 ± 1.3, (M) 1.9 ± 1.0, and (H) 2.1 ± 1.1 mL/100 mL/min; and TBV, (L) 5.7 ± 2.1, (M) 4.3 ± 1.9, and (H) 3.9 ± 1.2 mL/100 mL, respectively. Intratumoral hypoxic regions (M and H) showed significantly lower TBF, TMRO_2_, and TBV values than non-hypoxic regions (L). OEF showed significant increase in the severe hypoxic region compared to non-hypoxic and mild hypoxic regions.

**Conclusions:**

This study demonstrated that intratumoral hypoxic regions showed decreased blood flow with increased oxygen extraction, suggesting the need for a treatment strategy to normalize the blood flow for oxygen-avid active tumor cells in hypoxic regions.

**Electronic supplementary material:**

The online version of this article (doi:10.1186/s13550-017-0263-6) contains supplementary material, which is available to authorized users.

## Background

It is well known that the hypoxic regions in tumors can be resistant to radiotherapy as well as chemotherapy [1]. PET imaging of a hypoxic tracer, such as ^18^F-fluoromisonidazole (FMISO), can be used to detect hypoxia in patients with head and neck cancer and predict an adverse prognosis against hypoxia-targeting agents [2]. ^18^F-FMISO is a 2-nitroimidazole, which accumulates in the tissue environment at low oxygen concentrations by binding to macromolecules after reductive activation [3]. The rate of its accumulation increases as partial pressure of oxygen (pO_2_) falls below 10 mmHg [4]. Clinical hypoxia thresholds based on pO_2_ measurements (2.5 and 5.0 mmHg) corresponded to the ^18^F-FMISO standardized uptake value (SUV) (2.0 ± 0.6 and 1.4 ± 0.5, respectively) [5]. However, ^18^F-FMISO PET cannot reflect regional oxygen metabolism, and in vivo quantitative imaging of oxygen consumption in the hypoxic tumor has not been investigated.

Tumor hypoxia is divided into two types, acute hypoxia and chronic hypoxia. Acute hypoxia is the result of pronounced heterogeneity of the tumor blood flow distribution, whereas chronic hypoxia is observed at the limits of oxygen diffusion (100–200 μm) away from blood vessels [6]. It has been suggested that intratumoral oxygen metabolism is heterogeneous depending on the tumor microenvironment and quantitative measurement of oxygen consumption is essential to elucidate the mechanism of tumor hypoxia as it is the shortage of oxygen supply by blood flow against oxygen demand. In the hypoxic regions, two conditions were assumed, one is the shortage of blood supply with reduced blood flow and maintained oxygen consumption; the other is increased oxygen demand with a maintained blood flow and increased oxygen consumption. In this study, we aimed to evaluate the relationship between intratumoral oxygen demand and blood supply in hypoxic regions by in vivo ^15^O-labelled gases and ^18^F-FMISO PET using the rat xenograft model of C6 glioma, focusing on the oxygen extraction.

## Methods

### Preparation of ^18^F-FMISO and ^15^O-labelled gases

^18^F-FMISO was produced by the method as described previously with minor modifications [7, 8]. In brief, ^18^F-fluoride was produced with a cyclotron by the ^18^O(p, n)^18^F nuclear reaction using ^18^O–H_2_O. ^18^F-fluoride was trapped with an ion-exchange resin QMA sep-pak light cartridge and eluted from the resin by 210 mmol/L K_2_CO_3_ solution (0.2 mL) and 31 mg/mL Kryptofix 2.2.2 in acetonitrile solution (K222, 0.7 mL). Water was removed by azeotropic distillation. To the residue, repeated addition of acetonitrile solution (0.2 mL) and azeotropic distillation was performed. The dried ^18^F-KF/K222 complex mentioned above was added to the precursor (3-(2-nitroimidazol-1-yl)-2-*O*-tetrahydropyranyl-1-*O*-toluenesulfonylpropanediol 5 mg) in acetonitrile solution (0.7 mL) and reacted at 110 °C for 10 min. After cooling, the reaction mixture was added to 0.5 M HCl (0.7 mL). The mixture was hydrolyzed by heating for 5 min at 100 °C. After cooling, the mixture was neutralized with 1.5 M sodium acetate and transferred to the high-pressure liquid chromatography (HPLC) injector. Crude product was purified with semi-preparative HPLC (column: YMC-ODS-AM 10 × 250 mm, mobile phase: 3% EtOH/H_2_O, flow rate: 5.0 mL/min). The purified fraction was evaporated to dryness, and the residue was dissolved in saline containing benzyl alcohol (24 μL). The specific activity ranged from 30 to 300 GBq/μmol at the end of synthesis. Radiochemical purity was higher than 98%.

^15^O-labelled gases were produced by a ^14^N(p, n)^15^O nuclear reaction with 2.0% O_2_ (for ^15^O–CO and ^15^O–O_2_) or 2.0% CO_2_ (for ^15^O–CO_2_) added N_2_ gas target at a 12-MeV proton 7 μA current accelerated by the CYPRIS^R^ HM 12S in-house cyclotron (Sumitomo Heavy Industry).

### Animal preparation and implantation of C6 glioma cells

Male Fischer rats from Charles River Japan, Inc. were housed under a 12-h light/12-h dark cycle and had free access to food and water. A rat glioma C6 cell line, which was derived from gliomas induced by *N*-nitrosomethylurea, was provided by the RIKEN BRC. The cells were cultured in MEM medium (Sigma-Aldrich) with 10% fetal bovine serum (Sigma-Aldrich) at 37 °C in a humidified incubator containing 5% CO_2_. Cultured cells were collected after washing in PBS and harvested with trypsin. Tumor xenograft models were established by the subcutaneous injection of 1 × 10^6 tumor cells suspended in 0.2 mL of culture medium and Matrigel (1:1; BD Biosciences) into the bilateral shoulder region of F344 rats (a total of two sites in the left and right shoulder per one rat). PET experiments were performed 2 to 3 weeks after the implantation of C6 glioma cells, when the tumor diameter had reached 1–2 cm.

### ^18^F-FMISO PET

Ten rats with tumor xenografts (8 weeks age, body weight = 220 ± 15 g) were anesthetized with 2% isoflurane with room air, and a Terumo 24-G indwelling cannula was inserted into the tail vein. PET/CT data were acquired with a small-animal PET system (Inveon PET/CT system, Siemens Medical Solutions) [9]. The animals were placed in a feet-first supine position in the PET scanner. ^18^F-FMISO (69.9 ± 10.1 MBq) was administered intravenously via the catheter cannula, and dynamic PET scans (60 min) were started at the same time (*n* = 4). Delayed static scans (10 min) were performed at 3 h post injection in all rats (*n* = 10). CT acquisition was performed before or after the PET acquisition.

### ^15^O-labelled gases inhalation PET

The next day after FMISO PET, the tumoral blood flow (TBF), tumoral metabolic rate of oxygen (TMRO_2_), oxygen extraction fraction (OEF), and tumoral blood volume (TBV) were measured with a steady-state inhalation method under artificial ventilation of ^15^O–CO_2_, ^15^O–O_2_, and ^15^O–CO gases mixed with room air and oxygen [10, 11]. Rats were anesthetized with 2% isoflurane with room air during the arterial cannulation in the femoral artery, later switched to intramuscular injection of midazolam (1.2 mg/kg body weight), xylazine (4.8 mg/kg body weight), and butorphanol (1.6 mg/kg body weight). The stability of the anesthesia was already established in the previous study [10]. Tracheotomy was performed, and artificial ventilation was started (tidal volume 2–3 mL, frequency 60/min).

Inhalation of the ^15^O–CO_2_ (200 MBq/min) or ^15^O–O_2_ (400 MBq/min) gas was continued during the PET measurements for 16 min. The inhalation time of the ^15^O–CO gas (400 MBq/min) was 3 min, and the PET measurements were continued for a total of 13 min. Arterial blood sampling (0.1 mL) was performed during the steady-state PET acquisition in the ^15^O–CO_2_ and ^15^O–O_2_ gases studies (13 and 16 min after the start of the scanning) and 10 min after the start of the scanning in the ^15^O–CO gas study (A total of five blood samplings per rat). The radioactivity and weight of the whole blood and plasma after centrifugation (3000 round/min (800*g*), 3 min) were measured with a well scintillation counter (BeWell, Molecular Imaging Labo). CT acquisition was performed after the PET acquisition for the attenuation and scatter correction.

Systemic blood pressure (BP) and heart rate (HR) were indirectly measured with a tail-cuff apparatus (BP-98A-L, Softron). Arterial blood gas (ABG) analysis was performed from the blood samples (i-STAT system, Abbott Point of Care Inc). The body temperature was maintained with a heating sheet system.

### Quantitative data calculation

All PET data were reconstructed with 3-dimensional ordered-subset expectation maximization followed by maximum a posteriori (OSEM3D-MAP) (16 subsets, 2 OSEM3D, and 18 MAP iterations) with attenuation and scatter correction. The image matrix was 128 × 128 × 159, which yielded a voxel size of 0.776 × 0.776 × 0.796 mm. Quantitative values of TBF, TMRO_2_, OEF, and TBV were calculated by the steady-state inhalation method by Frackowiak et al. [10, 11]. Detailed calculation formulas were written in our previous study [10]. In brief, TBF was calculated from the ^15^O–CO_2_ PET using the radioactivity counts of the whole blood during the steady state. TMRO_2_ and OEF were calculated from the ^15^O–CO_2_ and ^15^O–O_2_ PET using the radioactivity counts of the whole blood and plasma during the steady state. TBV was calculated from the ^15^O–CO PET using the radioactivity counts of the whole blood. Functional images of the TBF, TMRO_2_, OEF, and TBV were reconstructed using in-house software from the last 6-min frame of each ^15^O-labelled gases study. A tissue–blood partition coefficient for water was fixed at 0.91 mL/g [12]. The correction value of the hematocrit between the great vessels and the tissue was fixed at 0.70 [13]. The TBV data were used to correct for intravascular hemoglobin-bound ^15^O_2_ [14].

### Quantitative PET data analysis

^18^F-FMISO PET images were co-registered to ^15^O-labelled gases PET with reference to the CT image using AMIDE software (Ver. 1.0.1) and PMOD software (Ver. 3.404). To compare the distribution of the two PET images, multiple small volumes of interest (VOIs, 1-mm sphere) were manually placed over the tumor of ^18^F-FMISO PET images (static images 3 h post injection) and functional images of ^15^O-labelled gases PET according to a previous study [15]. In setting VOIs, co-registered coronal images of ^18^F-FMISO (window level 0–3) and TBF (window level 0–80) were displayed using view tool of PMOD and 1-mm sphere VOIs were paved on PET images over the entire tumor where there is an uptake of ^18^F-FMISO or TBF (Additional file 1: Figure S1).

In addition, 8-mm sphere VOIs were placed on the forelimb muscle and used for the evaluation of background tissue activity to determine the threshold level of significant FMISO uptake. The quantitative values of TBF, TMRO_2_, OEF, and TBV were plotted against the ^18^F-FMISO SUV for correlation analysis. Next, VOIs were divided into three groups according to the ^18^F-FMISO SUV: group Low, low ^18^F-FMISO uptake region (less than 1.0 in SUV); group Medium, moderate ^18^F-FMISO uptake region (between 1.0 and 2.0 in SUV); and group High, high ^18^F-FMISO uptake region (more than 2.0 in SUV). The threshold value between group Low and Medium was determined by the background tissue activity, using the forelimb muscle uptake of ^18^F-FMISO SUV (the value obtained by adding the average and twice the standard deviation). The threshold value between group Medium and High was determined according to the previous study which reported the ^18^F-FMISO SUV of 2.0 corresponded to the pO_2_ measurement of 2.5 mmHg [5].

### Immunohistochemistry

After ^15^O-labelled gases PET/CT, pimonidazole (hypoxia marker, HypoxyprobeTM-1, 60 mg/kg) was injected 60 min before euthanasia. The tumor tissue samples were resected and fixed overnight with 4% paraformaldehyde and later cryoprotected in 30% sucrose in PBS before storage at −80 °C. Frozen sections (10–14 μm thick) were obtained with a cryostat (CryoStar NX70, Thermo Scientific) and mounted on glass slides. After blocking with blocking agent (Dako REAL Peroxidase-Blocking Solution) for 5 min at room temperature, incubations with the primary antibody against pimonidazole (FITC-MAb1, 1/100 dilution) for 30 min and the second antibody (HRP linked to rabbit anti-FITC, 1/100 dilution with Dako REAL Antibody Diluent) for 30 min were performed in order. Mounted sections were coverslipped after dehydration in ethanol and clearance with xylene, followed by 3,3′-Diaminobenzidine (DAB) (Dako Envision) and hematoxylin staining.

### Statistical analysis

Correlation analyses of TBF, TMRO_2_, OEF, and TBV against the ^18^F-FMISO SUV were performed with Spearman’s correlation coefficient. The quantitative values of TBF, TMRO_2_, OEF, and TBV were compared among the three groups with multiple comparison tests (Games–Howell test). Probability values of less than 0.05 were considered to denote statistical significance using SPSS version 19.0 (SPSS).

## Results

**Fig. 1:**
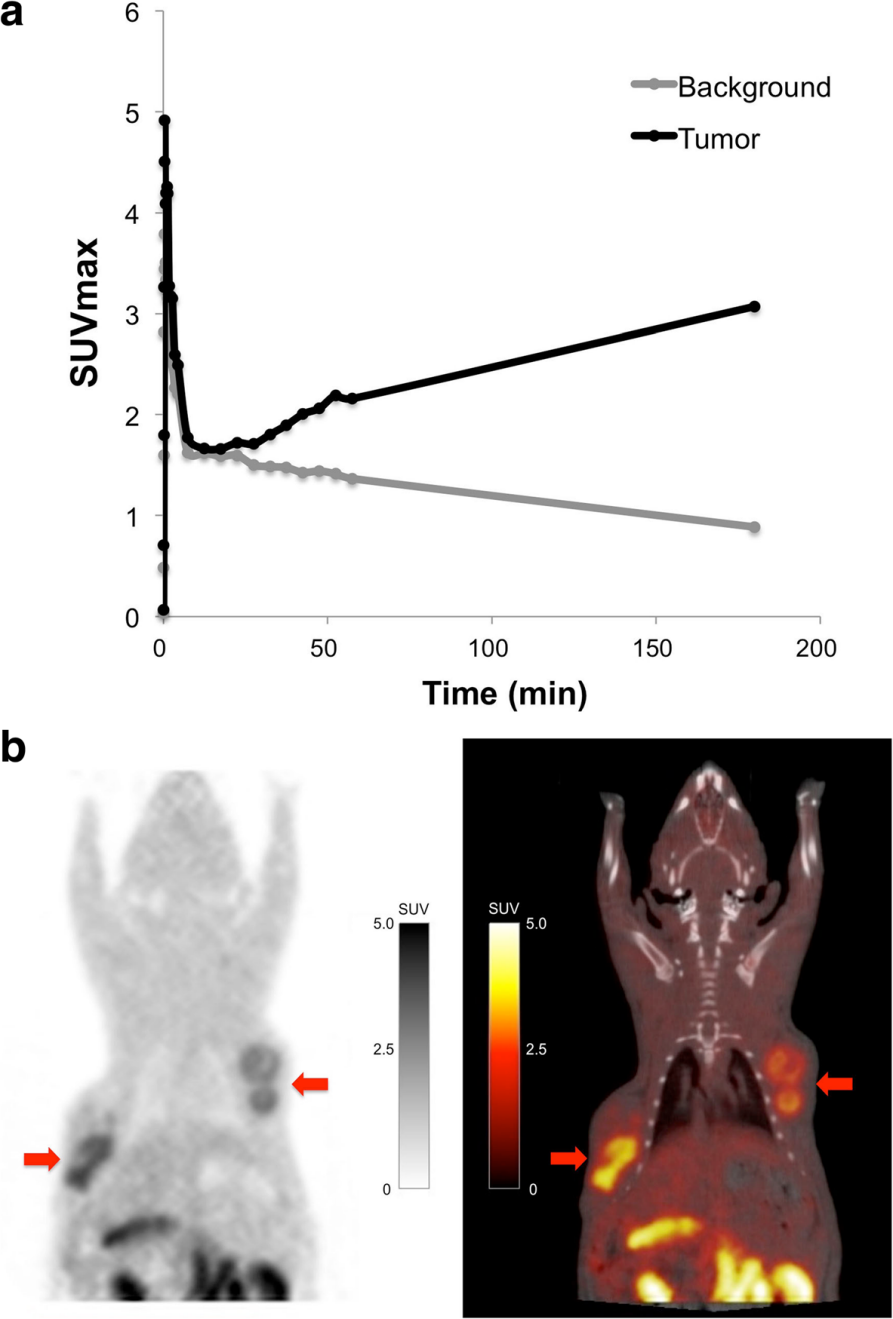
**a** The time activity curves of maximum SUV in the tumor and background tissue of ^18^F-FMISO PET. **b** Coronal images of ^18^F-FMISO PET and PET/CT fusion (3 h post injection). Tumors are located in the bilateral shoulder region of rats (*red arrows*)

The number of animals and tumor regions which were included in the analysis are summarized in Table 1. The time activity curves of ^18^F-FMISO PET showed increased accumulation in the tumor and wash out from background tissue over time (Fig. 1a). Hypoxic regions in the tumor were clearly visualized at 3 h post injection of ^18^F-FMISO (Fig. 1b).

**Table 1:** Number of animals and tumor regions which were included in the analysis

PET	Animal	Tumor
Dynamic ^18^F-FMISO PET (60 min)	4	8
Static ^18^F-FMISO PET (3 h post injection)	10	20
^15^O–CO_2_ PET (TBF)	10	20
^15^O–O_2_ PET (TMRO_2_ and OEF)	9	18
^15^O–CO PET (TBV)	10	20

In one rat, TMRO_2_ and OEF images were not generated and excluded from the evaluation due to the technical reason

**Fig. 2:**
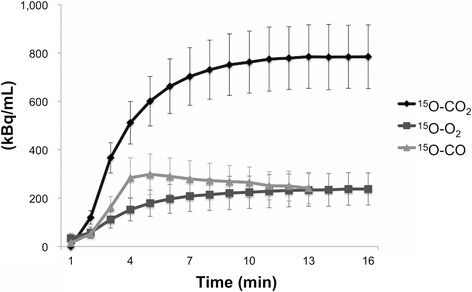
The time activity curves (mean ± standard deviation) of whole tumor of ^15^O-labelled gases PET (^15^O–CO_2_ and ^15^O–O_2_ gases, radioactivity count without decay correction; ^15^O–CO gas, radioactivity count with decay correction)

**Fig. 3:**
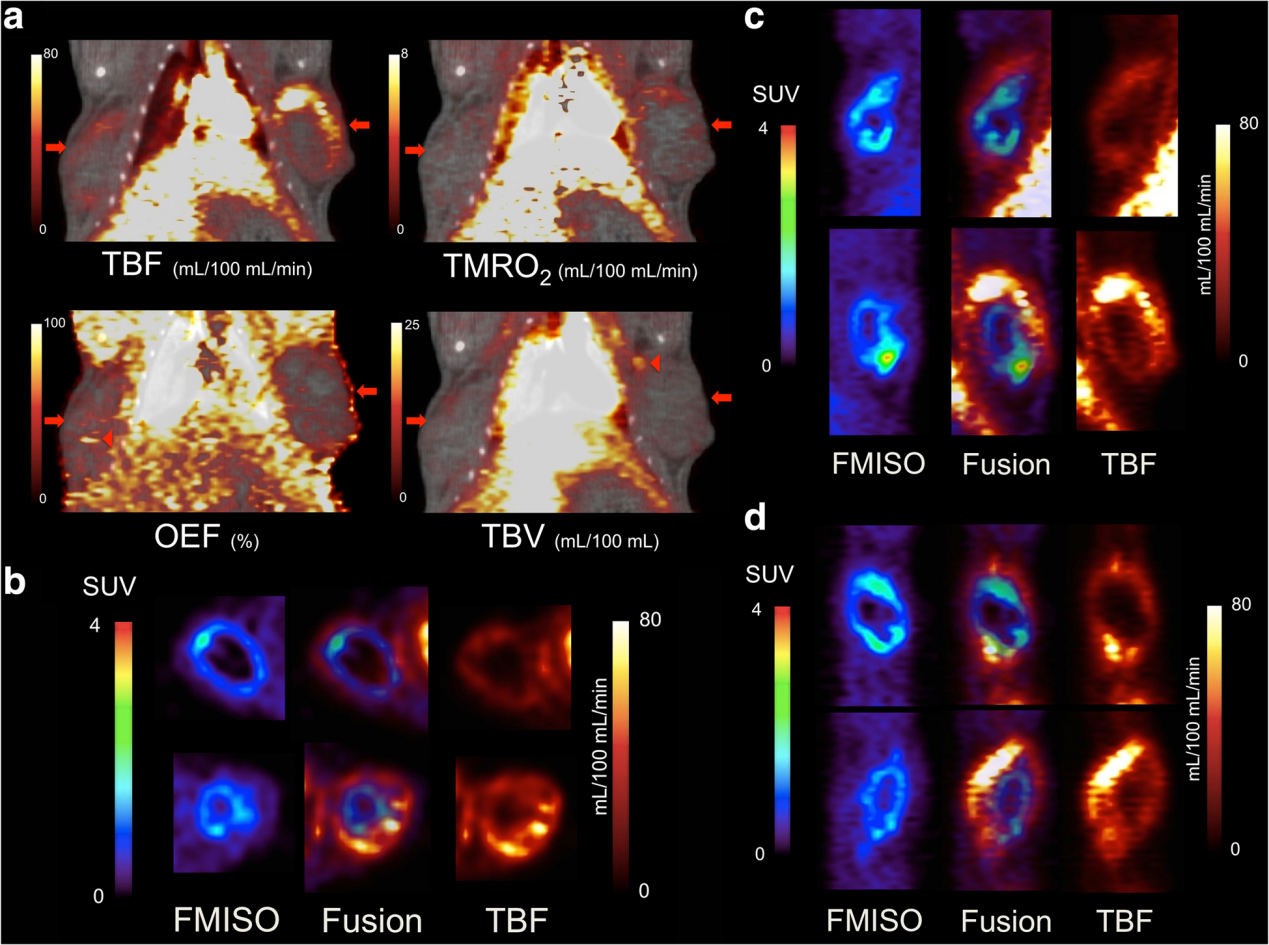
**a** Functional images of ^15^O-labelled gases PET using 6-min data during steady-state acquisition (coronal slices of PET/CT fusion). TBF and TMRO_2_ images showed marginal dominant distribution (*arrow*). OEF images showed partial elevation in the area close to the center (*arrow head*) and TBV images showed a high spot in the marginal zone of the tumor (*arrow head*). **b** Axial, **c** coronal, and **d** sagittal images of ^18^F-FMISO PET (*rainbow color scale*), TBF PET (*fire color scale*), and co-registered fusion images. The upper figures are tumors in the right side and the lower figures are tumors in the left side

The mean systolic and diastolic BP, HR, PaO_2_, PaCO_2_, hemoglobin concentration (Hb), hematocrit (Ht), and oxygen saturation (SaO_2_) during ^15^O-labelled gases PET are summarized in Table 2. The time activity curves of ^15^O-labelled gases PET reached a steady state approximately 10 min after continuous inhalation of ^15^O–CO_2_ or ^15^O–O_2_ gas (Fig. 2). Figure 3a shows mild but almost the same distribution pattern in TMRO_2_ as compared to TBF, partial elevation of OEF in the area close to the center, and a high TBV spot in the marginal zone of the tumor. Co-registered images of ^18^F-FMISO and TBF showed that intratumoral hypoxic regions with FMISO uptakes were located as lining the inside of the high TBF area and there was no accumulation in the central area inside the FMISO uptake, suggesting a necrotic region (Fig. 3b–d).

**Table 2:** Mean ± standard deviation values of BP, HR, and arterial blood gas data during PET measurement

Systolic/diastolic BP (mmHg)	119 ± 14/90 ± 10
HR (bpm)	379 ± 16
pH	7.420 ± 0.027
PaCO_2_ (mmHg)	42.4 ± 3.2
PaO_2_ (mmHg)	113.3 ± 30.2
SaO_2_ (%)	97.1 ± 2.1

**Fig. 4:**
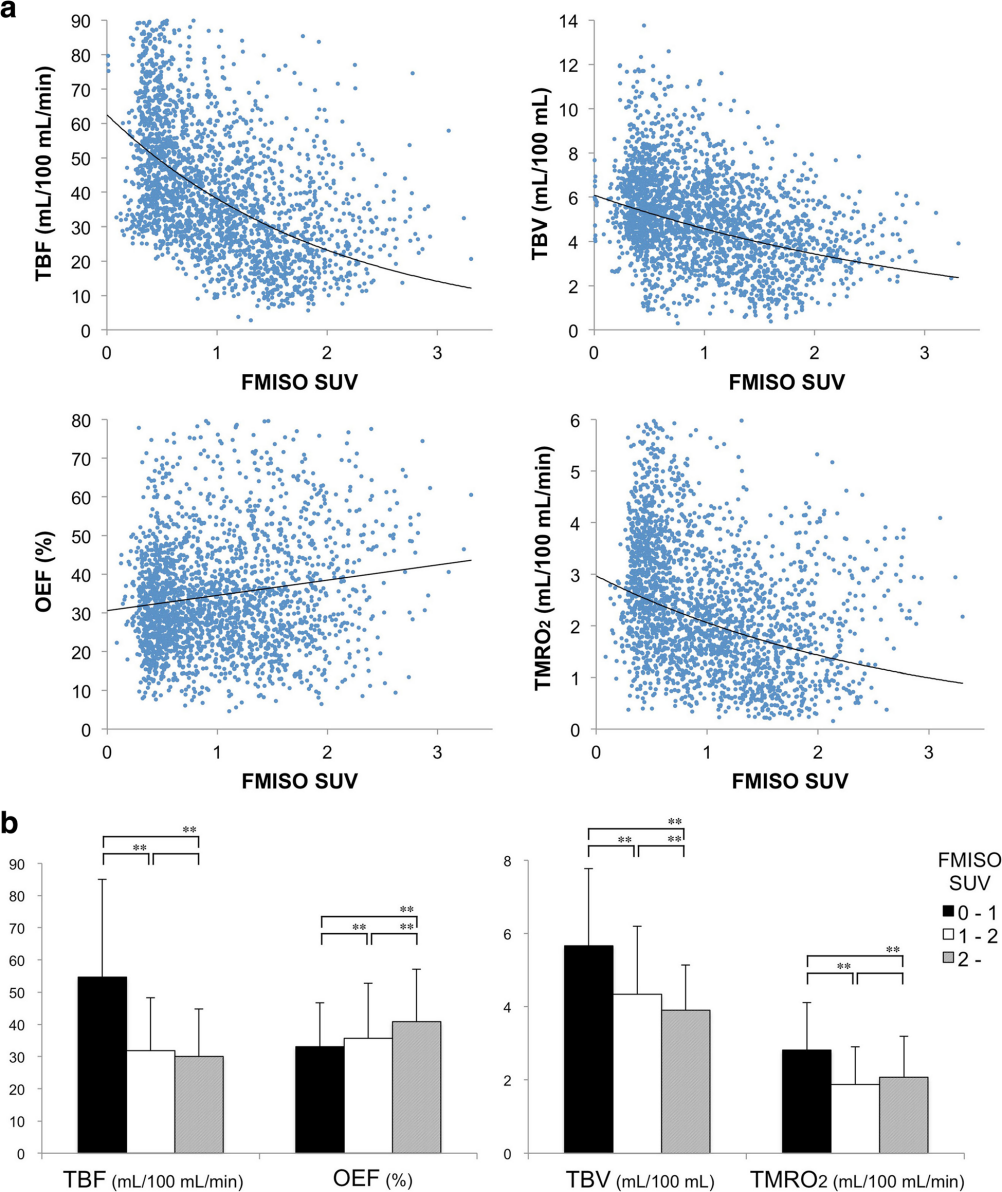
**a** Relationships between the ^18^F-FMISO SUV and quantitative values of TBF, TMRO_2_, OEF, and TBV. **b** Quantitative values (mean ± standard deviation) of group Low with less than 1.0 in ^18^F-FMISO SUV, group Medium with between 1.0 and 2.0 in ^18^F-FMISO SUV, and group High with more than 2 in ^18^F-FMISO SUV (**p < 0.01 with the multiple comparison test)

Relationships between ^18^F-FMISO uptake and quantitative values of ^15^O-labelled gases PET are shown in Fig. 4a. There were moderate negative correlations between ^18^F-FMISO SUV and TBF (*r* = −0.56 and *p* < 0.01), and weak negative correlations between ^18^F-FMISO SUV and TMRO_2_ (*r* = −0.38 and *p* < 0.01) and ^18^F-FMISO SUV and TBV (*r* = −0.38 and *p* < 0.01). Quantitative values in the Low (L), Medium (M), and High (H) groups were as follows: TBF, (L) 55 ± 30, (M) 32 ± 17, and (H) 30 ± 15 mL/100 mL/min; OEF, (L) 33 ± 14, (M) 36 ± 17, and (H) 41 ± 16%; TMRO_2_, (L) 2.8 ± 1.3, (M) 1.9 ± 1.0, and (H) 2.1 ± 1.1 mL/100 mL/min; and TBV, (L) 5.7 ± 2.1, (M) 4.3 ± 1.9, and (H) 3.9 ± 1.2 mL/100 mL, respectively (Fig. 4b). Intratumoral hypoxic regions (group Medium and High) showed significantly lower TBF, TMRO_2_, and TBV values than non-hypoxic regions (group Low). OEF showed significant increase in the severe hypoxic region compared to non-hypoxic and mild hypoxic regions. These findings suggested that oxygen demand was relatively maintained by increased oxygen extraction in the hypoxic region.

**Fig. 5:**
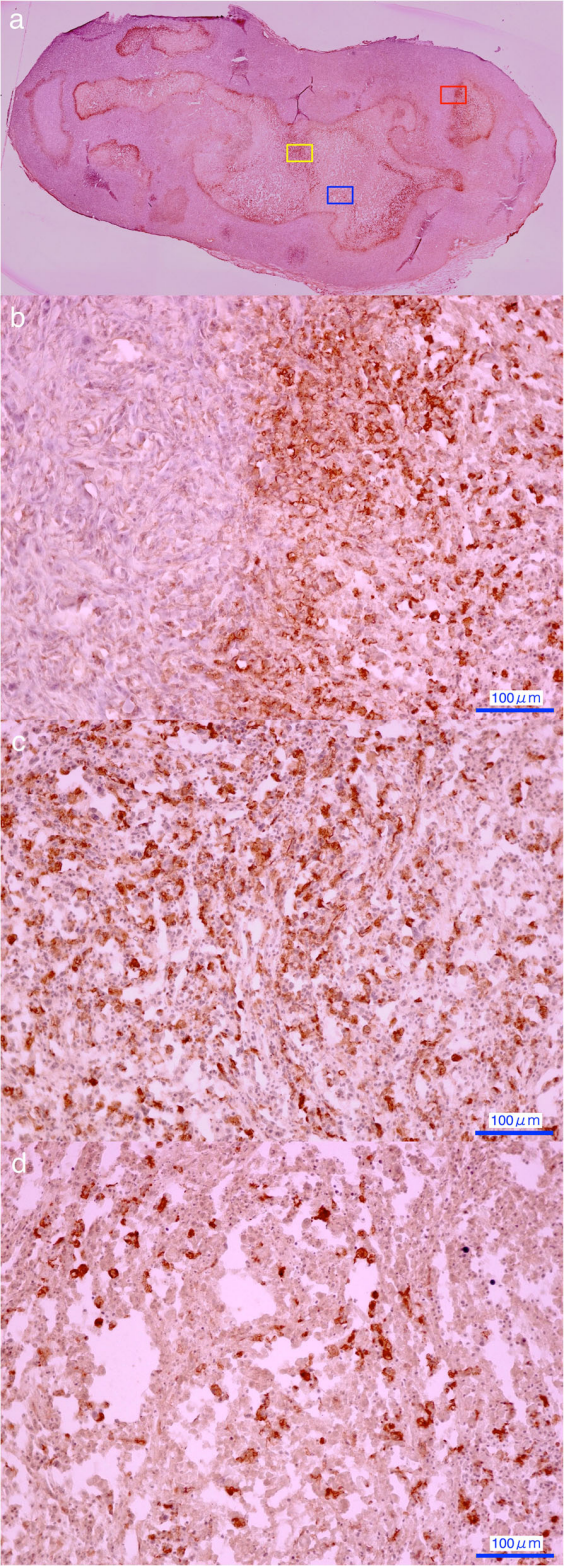
Immunohistochemistry of the intratumoral hypoxic region (pimonidazole staining). **a** Overall image of the tumor (summed image of ×20 magnification), **b** boundary zone: *red color square*, **c** hypoxic region: *yellow color square*, and **d** necrotic region: *blue color square* (×200 magnification)

**Fig. 6:**
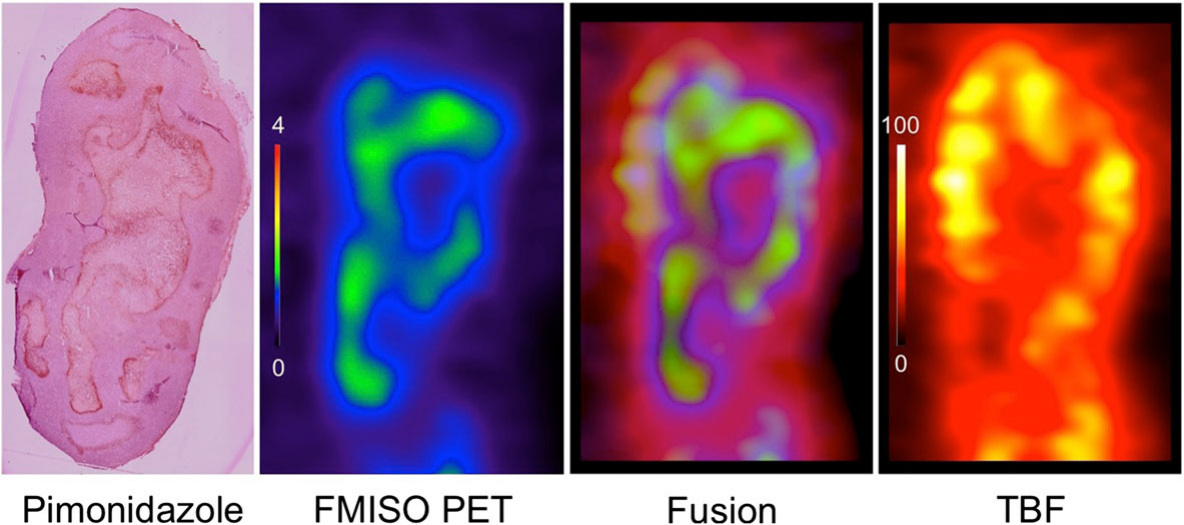
Comparison among pimonidazole staining, ^18^F-FMISO PET, TBF, and fusion image. Co-registered ^18^F-FMISO and TBF images were resliced to the same section as pimonidazole staining for comparison using the PMOD software

The pimonidazole-staining immunohistochemistry revealed a heterogeneous intratumoral distribution of the hypoxic regions, and high intensity staining was observed mainly at the edge of the viable region of the tumor section (Fig. 5a–c). The central area of the tumor showed necrotic regions partially accompanied by hypoxic cells (Fig. 5d). Pimonidazole staining corresponded to ^18^F-FMISO uptake regions, surrounded by blood flow preserved regions (Fig. 6).

## Discussion

We have reported herein on the relationship between blood supply and oxygen consumption in intratumoral hypoxic regions according to the ^18^F-FMISO SUV. Our results indicated that tumor cells in the hypoxic environment try to maintain the oxygen metabolism against decreased blood supply similar to the misery perfusion which has been observed in steno-occlusive disease in the brain [10, 16]. As shown in Fig. 4, we observed wide range of TBF or TMRO_2_ values in the tumors. This variability is considered to result from intertumoral heterogeneity. In some tumors, we observed decreased trend of TBF and TBV, increased trend of OEF, and stable TMRO_2_ against the increase of ^18^F-FMISO SUV (Additional file 1: Figure S2). However, weak correlation was observed between OEF and ^18^F-FMISO SUV as a whole, possibly due to the intertumoral heterogeneity of blood flow and oxygen utility level. Figure 4b shows significant increase of OEF along with increased ^18^F-FMISO SUV, suggesting that high ^18^F-FMISO uptake regions could survive the hypoxic conditions by the mechanism of elevated OEF.

Previous studies have reported perfusion and oxygen metabolism in patients with cerebral gliomas using ^15^O-labelled gases PET [17–20]. The TBF and TBV values varied widely depending on the vascularity of the tumor, whereas significantly decreased TMRO_2_ and OEF values were observed in the tumor region compared with normal gray matter or white matter in the brain. Compared to the normal brain tissue, glioma cells demanded less oxygen and received excessive TBF, which has been defined as the condition of luxury perfusion. The authors concluded that the mechanism of this luxury perfusion is unknown, whether it was due to decreased oxygen consumption as a result of adaptation to hypoxic conditions or was inherent in the metabolism of the gliomas. In our study, relatively increased OEF was observed in hypoxic regions against non-hypoxic regions, but OEF was decreased compared to the cerebral OEF (65 ± 9.1%) in the normal rat brain according to our previous study [10]. This result was consistent with the previous study in human by Mineura et al., which reported significantly decreased OEF in the tumor compared to the normal brain. The new findings of our study was that intratumoral hypoxic regions showed relatively increased oxygen extraction as well as decreased blood flow compared to the non-hypoxic regions.

We selected ^18^F-FMISO for a hypoxic tracer as it had been used most extensively in the previous study [21]. ^18^F-FMISO PET significantly correlated with the pimonidazole immunohistochemical staining and the endogenous hypoxia related marker CA IX [22]. ^18^F-FMISO has no protein binding, diffuses freely, and accumulates in those regions with a low oxygen concentration, which enables a delayed scan (2–3 h post injection) to achieve the best contrast [23, 24]. We compared the two PET scans in this study, and multiple VOI-based comparison was a feasible method as reported in the previous study to prevent high variability of data in the voxel based analysis [15]. VOI size of 1 mm was selected because intratumoral distribution of ^18^F-FMISO was heterogeneous, and larger VOIs led to ambiguous evaluation between the hypoxic and edge regions. In ^15^O-labelled gases experiments, we need at least 2 h for total anesthesia time from the preparation of an arterial sampling line to the end of ^15^O-labelled gases PET. Performing ^15^O-labelled gases and FMISO studies in 1 day is an extreme overload for rats due to the prolonged anesthesia. We performed less invasive FMISO PET at first and set the interval of 1 day between FMISO and ^15^O-labelled gases PET in this study. The anesthesia methods were different between FMISO PET and ^15^O-labelled gases PET. We monitored the systemic blood pressure, heart rate, and arterial blood gas data during ^15^O-labelled gases PET and confirmed that these factors were within physiological range and stable (Table 2). In addition, pimonidazole staining after ^15^O-labelled gases PET matched with the ^18^F-FMISO PET. It was considered that the difference about anesthesia had a minor effect for the evaluation of tumor perfusion and oxygen metabolism.

As for the arterial measurement during ^15^O-labelled gases PET, two blood samples were taken per each scan during steady state (13 and 16 min after the start of the scanning) and the average value was used for the quantitative calculation. Percent differences of the radioactivity between the two samples were 4.5 ± 3.3% for the whole blood and 6.0 ± 5.6% for the plasma. The measurements of arterial blood were considered to be sufficiently accurate and stable.

There are two types of tumor hypoxia, acute hypoxia (perfusion limited hypoxia) and chronic hypoxia (diffusion limited hypoxia) [6, 21]. Acute hypoxia is a transient hypoxic condition which can change from day to day, whereas chronic hypoxia is a rather stable condition. There is a possibility that hypoxic regions could be different between ^18^F-FMISO PET and ^15^O-labelled gases PET. Okamoto reported the high reproducibility of tumor hypoxia by comparing two ^18^F-FMISO PET scans within a 48-h interval in patients with head and neck cancer [25]. They concluded that the values for the ^18^F-FMISO PET uptake (SUV and uptake ratio of tumor to muscle) and the hypoxic volume between the two scans were highly reproducible. Furthermore, we performed immunohistochemical staining with pimonidazole after ^15^O-labelled gases PET, which corresponded to the ^18^F-FMISO PET findings seen on the previous day. These results demonstrated that the main hypoxia in the tumor was chronic hypoxia. However, acute component cannot be excluded in the low TBF regions as we evaluated one time point evaluation of ^15^O-labelled gases PET. Further study is necessary to evaluate the involvement of acute ischemia by investigating the temporal change, such as repetitive ^15^O-labelled gases PET studies.

As shown in Figs. 3 and 5, the central regions in the tumors were deficient in ^18^F-FMISO uptake and blood flow, which corresponded to the necrotic regions. These regions were excluded from the VOIs analysis in this study as multiple small sphere VOIs were placed on the tumor with ^18^F-FMISO uptake or blood flow uptake. However, since some necrotic regions were mixed with viable hypoxic cells as shown in the pimonidazole staining images, it is not always possible to clearly separate the hypoxic and necrotic regions in the histopathology.

This study demonstrated that tumor cells were resistant to the reduction in blood flow through the increased OEF and it will be an effective treatment strategy to modulate the blood flow, such as therapy using bevacizumab with a monoclonal antibody targeting vascular endothelial growth factor which normalizes vascular architecture, to deliver anti-tumor drugs more effectively and enhance the radiosensitizing effect through oxygenation. Bevacizumab is effective for some cancer patients in combination with chemotherapy. Recently, treatment with bevacizumab has been performed in patients with malignant gliomas, which led to an improved blood flow to the tumor [26, 27]. However, it was reported that high doses of bevacizumab might have the risk of increased hypoxia due to the decreased perfusion in the tumor [28]. Conversely, low dose could not yield survival benefit in non-small-cell lung cancer patients treated with 15 or 7.5 mg/kg bevacizumab with chemotherapy [29]. Another study also reported that decreased perfusion also resulted in the reduced drug delivery after administration of bevacizumab and scheduling of anti-angiogenic drugs should be optimized [30]. It was suggested that optimizing the individual dose could improve patient outcome by the pre-treatment evaluation with imaging techniques, such as FMISO and ^15^O-labelled gases or water PET, as perfusion and hypoxic conditions are heterogeneous in each cancer patient. In addition, there is no enough evidence about temporal change of perfusion and oxygen metabolism after bevacizumab therapy. The next step of our study is to elucidate how the oxygen metabolism could be changed after the vascular normalization with bevacizumab.

There are some limitations in this study. In small-animal PET systems, the spatial resolution (full width at half maximum, FWHM) of ^15^O is approximately doubled compared to ^18^F or ^11^C due to the longer positron range [31]. The spatial resolution is about 1.5 mm FWHM in this study, and the partial volume effect (PVE) might be larger on ^15^O-labelled gases PET images than ^18^F-FMISO PET (*9*). PVE is a principle problem of PET that has been long discussed. We must pay attention to the underestimation by PVE in the evaluation of intratumoral distribution with PET. However, in our previous study, elevated OEF with decreased cerebral blood flow was detected by ^15^O-labelled gases PET in the rat stroke model, suggesting the feasibility to detect the subtle changes in tumor perfusion or oxygen consumption. Next, we fixed the tissue–blood partition coefficient as 0.91 mL/g and the correction value of the hematocrit as 0.70 according to previous studies of the brain [12, 13]. If the partition coefficient was substantially different in some area, it has some influence on TBF values. However, these effects were relatively small in low TBF area, such as necrotic area. TBF was increased from 20 to 22 or 25 mL/100 mL/min when the partition coefficient was changed from 0.91 to 0.8 or 0.7, respectively. In addition, previous study reported tissue heterogeneity always resulted in underestimations of mean values of oxygen extraction fraction by the simulation [32]. It may be necessary to develop a new tracer kinetic model considering tumor heterogeneity for more accurate evaluation. Third, this study was conducted on a single tumor model without using anti-tumor drugs. It is the next challenge to investigate other types of tumor model including the relationship between glycolysis and oxidative phosphorylation and to evaluate the changes in perfusion and oxygen metabolism after the anti-tumor therapy or modulation of perfusion and hypoxia.

## Conclusions

This study demonstrated the in vivo relationships among ^18^F-FMISO SUV, TBF, TMRO_2_, OEF, and TBV values in the rat xenograft model of C6 glioma with an immunohistochemical evaluation. Intratumoral hypoxic regions showed decreased blood flow with increased oxygen extraction compared to the non-hypoxic regions. These findings suggested that a treatment strategy to normalize the blood flow would be a reasonable approach for oxygen-avid active tumor cells in hypoxic regions to achieve more effective drug delivery in chemotherapy and an oxygen sensitizing effect in radiation therapy.

## Additional file

Additional file 1: Figure S1. Multiple VOI settings on coronal fusion image of ^18^F-FMISO PET (window level 0–3 in SUV) and TBF PET (window level 0–80 ml/100 mL/min). VOIs (1-mm sphere) were manually placed over the entire tumor where there is an uptake of ^18^F-FMISO or TBF on the fusion PET images. **Figure S2.** Relationships between the ^18^F-FMISO SUV and quantitative values of TBF, TMRO_2_, OEF, and TBV of each tumor. Decreased trend of TBF and TBV, increased trend of OEF, and stable TMRO_2_ against the increase of ^18^F-FMISO SUV were observed. (DOC 673 kb)
