# Supplementary material for: Quantitative evaluation of oxygen metabolism in the intratumoral hypoxia: 18F-fluoromisonidazole and 15O-labelled gases inhalation PET
Source: EJNMMI Res. 2017 Feb 16;7:16. doi: 10.1186/s13550-017-0263-6 (PMC5313496; doi:10.1186/s13550-017-0263-6)
Supplement: Additional file 1: Figure S1. — Multiple VOI settings on coronal fusion image of 18F-FMISO PET (window level 0–3 in SUV) and TBF PET (window level 0–80 ml/100 mL/min). VOIs (1-mm sphere) were manually placed over the entire tumor where there is an uptake of 18F-FMISO or TBF on the fusion PET images. Figure S2. Relationships between the 18F-FMISO SUV and quantitative values of TBF, TMRO2, OEF, and TBV of each tumor. Decreased trend of TBF and TBV, increased trend of OEF, and stable TMRO2 against the increase of 18F-FMISO SUV were observed. (DOC 673 kb) [file 13550_2017_263_MOESM1_ESM.doc]

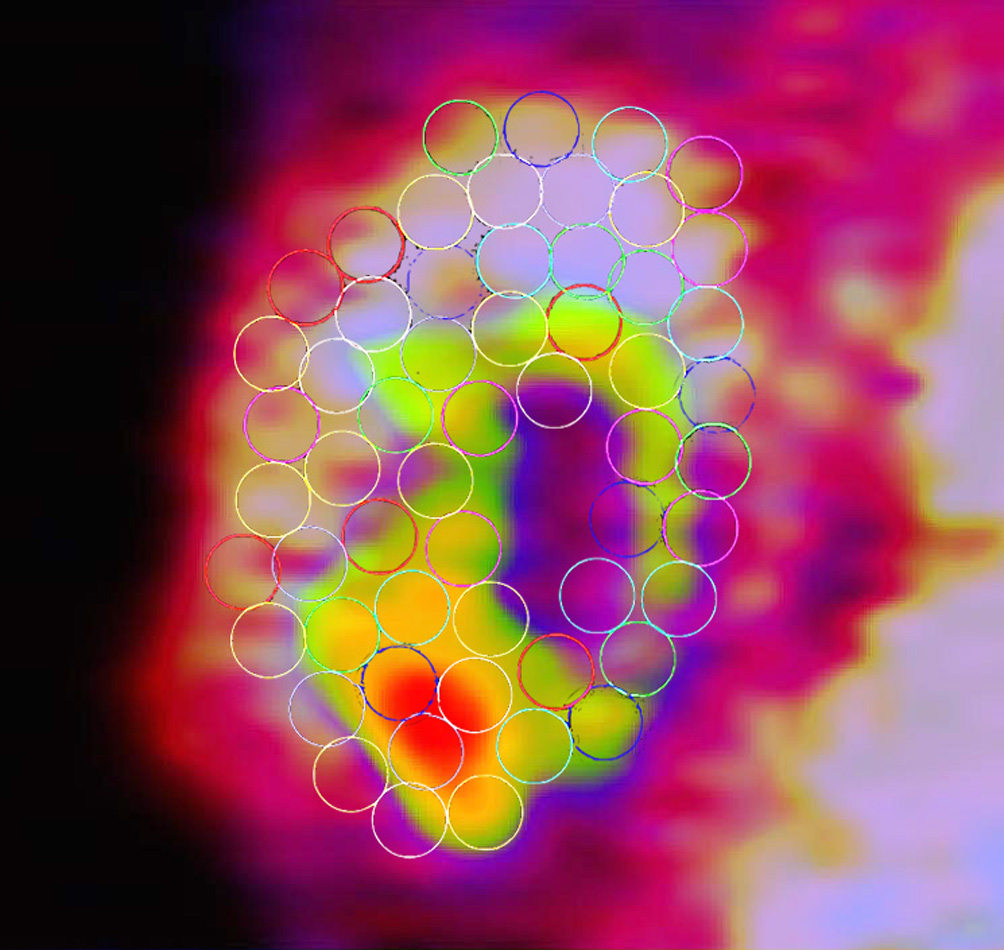


**Figure S1.** Multiple VOI settings on coronal fusion image of ^18^F-FMISO PET (window level: 0-3 in SUV) and TBF PET (window level: 0-80 ml/ 100mL/min). VOIs (1 mm sphere) were manually placed over the entire tumor where there is an uptake of ^18^F-FMISO or TBF on the fusion PET images.


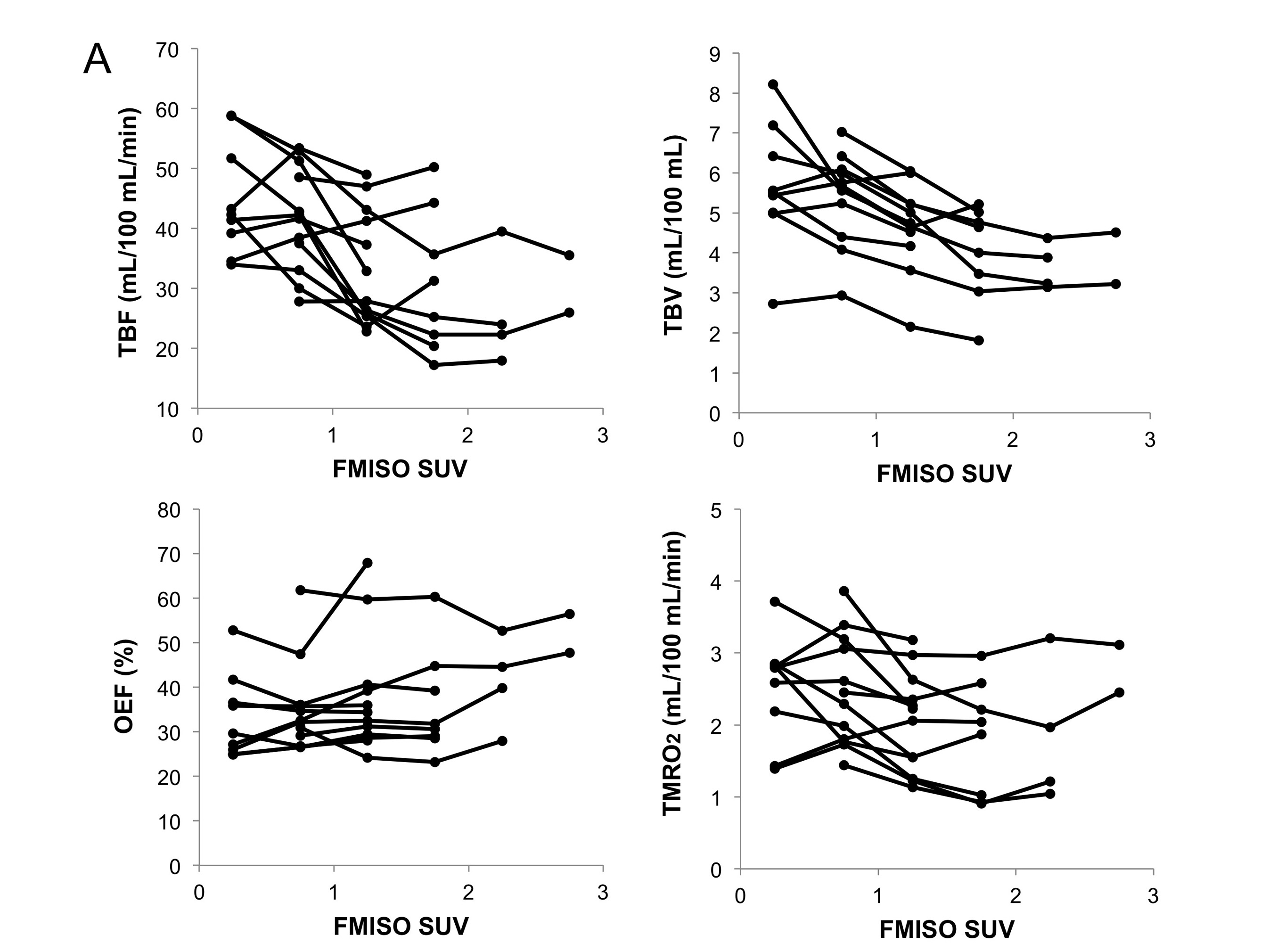


**Figure S2.** Relationships between the ^18^F-FMISO SUV and quantitative values of TBF, TMRO_2_, OEF, and TBV of each tumor. Decreased trend of TBF and TBV, increased trend of OEF, and stable TMRO_2_ against the increase of ^18^F-FMISO SUV were observed.
